# Human-in-the-Loop as a Safety Guardrail: Clinical Accountability in the Large Language Model Era

**DOI:** 10.2196/85726

**Published:** 2026-06-18

**Authors:** Isaac Zablah, Yolly Molina, Antonio Garcia-Loureiro

**Affiliations:** 1 Faculty of Medical Sciences, National Autonomous University of Honduras, Calle la Salud SN, Tegucigalpa, 11101, Honduras; 2 Center for Biomedical Imaging Diagnostics Research and Rehabilitation, National Autonomous University of Honduras, Tegucigalpa, Honduras; 3 Department of Electronics and Computer Science, Universidade de Santiago de Compostela, Santiago de Compostela, Spain

**Keywords:** large language models, high-performance computing, medical informatics, computational efficiency, clinical decision support, artificial intelligence, healthcare infrastructure, model optimization

We found Zhang et al’s thorough review of the transformative potential of large language models (LLMs) in healthcare to be very interesting [1]. The authors do a great job of talking about clinical applications, data integration, and ethical issues. However, we think that important aspects of computational performance need more attention, especially when it comes to using technology in real-world healthcare settings where resources are limited.

Zhang et al mention that “*technological advancements*” are helping to meet the “*high hardware requirements*” of LLMs [1], but the reality of computing is still a huge challenge. Modern medical LLMs such as GPT-4 and domain-specific models such as Med-PaLM 2 need considerable infrastructure as described below [2]:

- *Inference latency*: Currently, LLMs take 2 to 10 seconds to respond to each query. This may not be fast enough for clinical situations where time is of the essence, like triage in the emergency department or decision support during surgery. More detailed answers need more time [3].
- *Memory footprint*: Models with billions of parameters need 16-80+ GB of VRAM (video random access memory) for fast inference [4]. This means that many health care facilities, especially in low- and middle-income countries, do not have the specialized GPU infrastructure they need.
- *Scalability challenges:* Serving hundreds of concurrent clinical users requires distributed computing architectures and load-balancing strategies not discussed in the review [5].

For edge computing and improving models, we suggest that subsequent research should emphasize:

- *Model quantization and pruning:* Techniques to reduce model size by 50%‐75% with minimal accuracy loss, enabling deployment on consumer-grade hardware.
- *Edge computing solutions:* Local deployment using optimized models (eg, 7-13B parameter variants) to address data privacy concerns while reducing latency and cloud dependency.
- *Hybrid architecture:* Combining lightweight edge models for routine queries with cloud-based full models for complex cases, optimizing the accuracy-efficiency trade-off.

The medical informatics community requires standardized metrics that assess not only diagnostic accuracy but also operations per diagnosis (computational cost), energy consumption per inference (environmental impact), and cost-effectiveness ratios (accuracy gained per dollar of infrastructure). We did an initial benchmarking of three LLMs on differential diagnosis tasks: Clinical Camel (LLaMA-2-13B), PMC-LLaMA 13B, and Meditron-3 (Qwen2.5-14B). We found that smaller, domain-specific models (~14 billion parameters fine-tuned on medical corpora) were able to achieve 85%‐90% of GPT-4’s diagnostic accuracy while using only about 15% of the computational resources, indicating considerable room for improvement.

We want high-performance computing research in medical artificial intelligence (AI) to help with clinical implementation. This research should set benchmarks for both computational performance and clinical accuracy, come up with optimization techniques that are specific to medical inference workloads, create reference architectures for deploying LLMs in different health care settings, and investigate federated learning strategies that let training happen without putting sensitive patient data in one place.

The transformative potential Zhang et al describe will only be realized if LLMs can be deployed efficiently and equitably across diverse health care environments. High-performance computing and medical informatics must advance in tandem to bridge the gap between research promise and clinical reality.

## Abbreviations

AI: artificial intelligence
LLM: large language model
VRAM: video random access memory
